# Measurement Invariance and Psychometric Analysis of Oxford Happiness Inventory Scale across Gender and Marital Status

**DOI:** 10.1155/2020/8906209

**Published:** 2020-06-21

**Authors:** Amin Mousavi, Zahra Sharafi, Abdolreza Mahmoudi, Hadi Raeisi Shahraki

**Affiliations:** ^1^Department of Educational Psychology and Special Education, College of Education, University of Saskatchewan, Canada; ^2^Department of Epidemiology and Biostatistics, School of Health, Zahedan University of Medical Sciences, Zahedan, Iran; ^3^Health Promotion Research Center, Zahedan University of Medical Sciences, Zahedan, Iran; ^4^Department of Islamic Education, Faculty of Medicine, Shiraz University of Medical Sciences, Shiraz, Iran; ^5^Department of Epidemiology and Biostatistics, School of Health, Shahrekord University of Medical Sciences, Shahrekord, Iran

## Abstract

**Background:**

The Oxford Happiness Inventory (OHI) is a self-report tool to measure happiness. A brief review of previous studies on OHI showed the lack of evaluation of OHI fairness/equivalence in measuring happiness among identified groups.

**Methods:**

To examine the psychometric properties and measurement invariance of the OHI, responses of 500 university students were analyzed using item response theory and ordinal logistic regression (OLR). Relevant measures of effect size were utilized to interpret the results. Differential test functioning was also evaluated to determine whether there is an overall bias at the test level.

**Results:**

OLR analysis detected four items across gender and two items across marital status to function differentially. An assessment of effect sizes implied negligible differences for practical considerations.

**Conclusions:**

This study was a significant step towards providing theoretical and practical information regarding the assessment of happiness by presenting adequate evidence regarding the psychometric properties of OHI.

## 1. Introduction

Happiness has been the ultimate goal of humans and superior to all other goals throughout history. Previous researches indicated that happiness is rated higher than all other personal values, and it is also a highly valued component of life quality. Although the early tendency of psychological research was to focus on mental illness and social or occupational disorders, interest in the positive dimensions of human life (e.g., well-being and happiness) was increased in the late 20th century; thus, because of this new desire, different measures have been developed to assess happiness [1] The most widely used and respected questionnaires which measure the happiness are Subjective Happiness Scale [2], Satisfaction with Life Scale [3], and Panas Scale [4]. These questionnaires reflect different definitions and perceptions of happiness. The Oxford Happiness Inventory (OHI) [5] is another happiness instrument which is one of the most appropriate scales possessing several vital characteristics for assessing happiness such as easy to administer and allows endorsements over an extended range, adequate number of items, internal reliability and validity, and developmentally appropriate.

The OHI was devised as a broad measure of personal happiness in the Department of Experimental Psychology of the University of Oxford in the late 1980s. The development of the scale and some of its statistical properties were reviewed by Argyle, Martin, and Lu (1995). The scale has been found to behave consistently and was used cross-culturally to compare students in Australia, Canada, the UK, and the USA [6]. The OHI has also been studied in different countries such as China, Iran, and Italia [7–9].

In the cross-cultural study, OHI questionnaires were completed by four samples of undergraduate students: 378 in the UK, 212 in the USA, 255 in Australia, and 231 in Canada. Their findings support internal consistency among students in those countries. Furthermore, there were no significant sex differences in scores on the Inventory in any of our English-speaking samples. Granted those findings, the OHI can be recommended for use as a trait-measure in studies among undergraduates in each of those cultures [6].

An Italian adaptation of the OHI was administered to 782 adolescents. Exploratory structural modeling was used, and the total scale and the subscales of the Italian adaptation of the OHI are coherent with regard to both psychometric criteria and psychological meaning. Their results also supported the validity of the Italian version of the OHI as an instrument for measuring positive psychological functioning in adolescence. The scale also showed adequate internal consistency values and strong measurement invariance across gender [8].

Using Chinese samples in 1997, Lu and Shih were examining the psychometric properties of Chinese Happiness Inventory (CHI) which was based on the OHI. 200 adults aged between 18 and 65 years old living in Taiwan completed this measurement. Their result showed a negative direct relation between neuroticism and happiness and a positive direct relation between social desirability and happiness [9].

Bayani [7] examined the reliability and preliminary evidence for validity of a Persian version of the OHI in 309 undergraduate students (161 women and 148 men). In this study OHI, the Satisfaction with Life Scale, the Beck Depression Inventory, and the Depression-Happiness Scale were completed by participant. Analyses indicated that the Persian version of the OHI Scale has reliability as a measure of well-being and provided some preliminary evidence of construct validity [7]

A brief review of these previous studies on OHI shows the lack of evaluation of OHI fairness/equivalence in measuring happiness among identified groups. Measurement equivalence, also known as differential item functioning (DIF), is an important part of the process of validating questionnaires to test whether the probability of responding to a specific item exhibits different statistical properties for different identifiable groups after controlling the construct being measured [10, 11]. Therefore, the goal of this study was to assess measurement equivalence of happiness by means of OHI across gender and marital status. For achieving this goal, we followed the analytical framework employed by Mousavi et al. 2019 [12].

## 2. Method

### 2.1. Sample

This study involved 500 university students (62.4% boys, 37.6% girls) in 2018. The participants were randomly selected by a two-stage random sampling technique from Shiraz University of Medical Sciences, Iran. At the first stage, five out of the eleven faculties were selected randomly, and then for each faculty, 100 students were selected through random sampling. After explaining the aim of the study, informed consent forms were signed by the students who expressed their willingness to participate. The mean (±standard deviation) age of participants was 21.3 ± 3.7 years.

### 2.2. Instrument

The Oxford Happiness Inventory (OHI) [5] is a 29-item, self-report instrument, which was devised as abroad measure of personal happiness, mainly for in-house use in the Department of Experimental Psychology of the University of Oxford in the late 1980s [13]. The inventory was developed as a multidimensional scale to measure happiness, following the design and format of the Beck Depression Inventory (BDI). The instrument consists of items with an ordinal and polytomous scoring scale numbered from 0 to 3, so that the total scores range from 0 to 87, with higher scores showing greater happiness [8, 13]. The validity and reliability of the Persian version of OHI have also been investigated in different studies, and it has been found to be acceptable [7].

### 2.3. Item Response Theory (IRT)

IRT was utilized to assess the dimensionality and psychometric properties of the OHI. The goodness of fit statistics were used to identify the best fitting polytomous IRT model among graded response model (GRM), generalized partial credit model (GPCM), and rating scale model (RSM). The indices were based on M2 statistic [14]. Additionally, a likelihood ratio test was used to statistically compare the fitted models. Finally, the OHI was analyzed based on the best fitting IRT model.

### 2.4. Differential Item Functioning (DIF) Detection

In recent years, DIF has been widely used to ensure the internal validity of psychological, educational, and medical quality of life test scores across different demographic variables such as gender (e.g., Zampetakis et al., 2017), ethnic background (e.g., Jones et al., 2016), age groups (e.g., Estabrook et al., 2015), and socioeconomic status (e.g., Thielemann et al., 2018). DIF assesses whether the probability of responding to a specific item is different for different groups after controlling the ability [10, 11]. There are two forms of DIF known as uniform and nonuniform. Uniform DIF is defined as a constancy of differences in the probability of correct answer for manifest group at all ability levels, and nonuniform DIF happens when the direction of such difference changes at some ability levels [11, 15]. Methodology reviews showed that there are several parametric and nonparametric statistical methods for investigating bias at item as well as test level [11, 16, 17]. Among all parametric and nonparametric methods, ordinal logistic regression (OLR) [18] approaches have received notable attention in applied researches [15, 19]. This model-based procedure is effective, easy to implement which can control additional categorical and continuous covariates which may confound the results of DIF analysis [19–23]. Detecting DIF with utilizing OLR is based on comparing three different nested models. The models as given by French and Miller (1996) have the following forms:

(1) $$\begin{array}{c} \eta_{i}=\ln\left[\frac{p\left(Y_{i}\leq k\;|\;g,\theta \right)}{1-p\left(Y_{i}\leq k\;|\;g,\theta \right)}\right]=\beta_{0}+\beta_{1}\theta +\beta_{2}g+\beta_{3}\left(g*\theta \right)\text{ Model }1\\ \eta_{i}=\ln\left[\frac{p\left(Y_{i}\leq k\;|\;g,\theta \right)}{1-p\left(Y_{i}\leq k\;|\;g,\theta \right)}\right]=\beta_{0}+\beta_{1}\theta +\beta_{2}g\text{ Model }2\\ \eta_{i}=\ln\left[\frac{p\left(Y_{i}\leq k\;|\;\theta \right)}{1-p\left(Y_{i}\leq k\;|\;\theta \right)}\right]=\beta_{0}+\beta_{1}\theta \text{ Model }3 \end{array}$$

Where *p*(*Y*_*i*_ ≤ *k*) is the probability of responding at or below category *k* to an item for the *i*th person, *θ* represents ability and it is measured by the total test score, *g* is a grouping variable, and *g* × *θ* represents the interaction between grouping variable and ability. The value of the difference in -2 log-likelihood of model 1 and model 3 can be used to detect uniform and nonuniform DIF simultaneously. This value can be compared to a chi-square distribution with two degrees of freedom. If this comparison yields a significant result, the item is flagged for DIF, and then, further investigations are needed to test whether there is uniform or nonuniform DIF. Comparison of models 1 and 2 is used to assess nonuniform DIF. Uniform DIF also exist when models 2 and 3 differ significantly [11, 15, 24, 25].

The effect of sample size on the significance testing and necessity of reporting the effect size have been well documented [26]. Several studies have shown that test score-based methods such as logistic regression (LR) are prone to Type I error rate inflation (Gómez-Benito, Hidalgo, & Padilla, 2009). Therefore, when conducting studies to detect and interpret DIF, it is particularly useful to include measures of effect size as it is not sensitive to the sample size. The use of effect size measures optimizes the decision to retain or exclude an item with DIF and also reduces the incidence of false positive outcomes. Additionally, the exclusion of items that have been falsely identified with DIF can have serious effects on the reliability and validity of measurement instruments [27, 28]. The measures of effect size for all DIF items as suggested by Jodoin and Gierl (2001) were computed. The measure is the difference between two pseudo *R* squared [29], of model 2 and model 1 for nonuniform DIF and the difference between two pseudo *R* squared of model 3 and model 2 for uniform DIF. According to Jodoin and Gierl (2001), the magnitude of DIF can be considered as negligible if the difference is less than 0.035, moderate if it is between 0.035 and 0.07, and large if it is more than 0.07. Thus, flagged items with negligible effect size values are not problematic [30].

For assessment of DTF of polytomous items, *ν*^2^ was calculated based on Penfield and Algina [31]. The magnitude of DTF can be considered as small if *ν*^2^ is less than 0.07, medium if it is between 0.07 and 0.14, and high if it is more than 0.14 [32, 33].

As in case of dichotomous items, item characteristics curves (ICC) of the item under investigation for the reference and focal groups can be used to depict DIF. Similarly, the item characteristic function (ICF) is good summary statistics for polytomous item especially in order to illustrate DIF. The ICF is defined as the sum of the expected scores over response categories for each item (Nering and Ostini, 2011). When we have an item with *m*_*j*_ categories, ICF can be defined as the following formula:

(2) $$\begin{array}{c} E\left(X_{j}\;|\;\theta \right)=\overset{m_{j}}{\underset{x=0}{\sum }}xp_{jx}\left(\theta \right) \end{array}$$

Where *p*_*jx*_(*θ*) is the probability of a score of *x* in the jth response category of item X.

In this study, three IRT models were compared by mirt package in R3.3.2. Two different OLR models were also estimated for detecting DIF among gender (female = 0; male = 1), and marital status (single = 0; married = 1) which were conducted using ORDINAL package in R3.3.2 [34]. Additionally, DTF analyses for polytomous items were computed, using DIFAS 5 [35].

The goodness of fit indices of GRM, RSM, and GPCM are summarized in Table 1. Both the M2 statistic and other criteria showed fairly acceptable goodness of fit, but the GRM was found to be the best-fitting model.

## 3. Result

### 3.1. Item Response Theory Analysis

**Figure 1:**
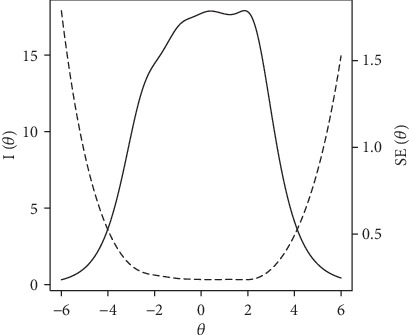
Test information function (solid curve) and its standard error (dash curve) for the whole test.

The goodness of fit between data and the three selected IRT models was assessed using fit indices and likelihood ratio test. Table 1 shows the goodness of fit indices for GRM, RSM, and PCM models. The M2 statistic and other fit indicate better fit between data and GRM model (RMSEA = 0.072, TLI = 0.927, and CFI = 0.933), but other models also seem to be appropriate. Thus, the likelihood ratio test of model was performed in search for any potential statistical difference among three models.  Table 2 shows a statistically significant difference between the three models despite having very close fit indices, and both GPCM and PCM models showed lower log-likelihood values with a trivial difference. Therefore, the OHI items were analyzed based on the GRM model as shown in Table 3. Regarding the item discrimination (i.e., in Table 3), all the items showed an adequate level of discriminant power ranging from 0.953 (for item 12) to 2.436 (for item 5) with an average discrimination power of 1.556. Regarding the item difficulties (i.e., *b* values in Table 3), there are three thresholds (i.e., *b*1, *b*2, and *b*3) for each item, since the item response is recorded based on a four-point Likert-scale. The first threshold reflects the least amount of the underlying attribute needed to endorse the first option, and the last threshold indicates the maximum level of the underlying attribute needed to endorse the last category. The threshold values showed an incremental trend with average values of -1.988, 0.213, and 2.297 for *b*1, *b*2, and *b*3, respectively. Goodness of fit with the GRM model at item level was examined by the polytomous extension of S-X^2^ [36] and are shown in Table 3. As shown only item 21 was identified as misfitting at *p* value <0.05. All other items showed acceptable fit to the GRM model. Test information function and standard error of measurement in OHI are shown in Figure 1. This graph shows that the OHI is more informative and precise in the middle range of the underlying attribute (i.e., values approximately between -2 and 2). This is congruent with the aim of this tool which is measuring happiness in a broad sense. The IRT analysis of OHI asserts its psychometric quality for measuring happiness.

### 3.2. Differential Item Functioning (DIF) Analysis

**Figure 2:**
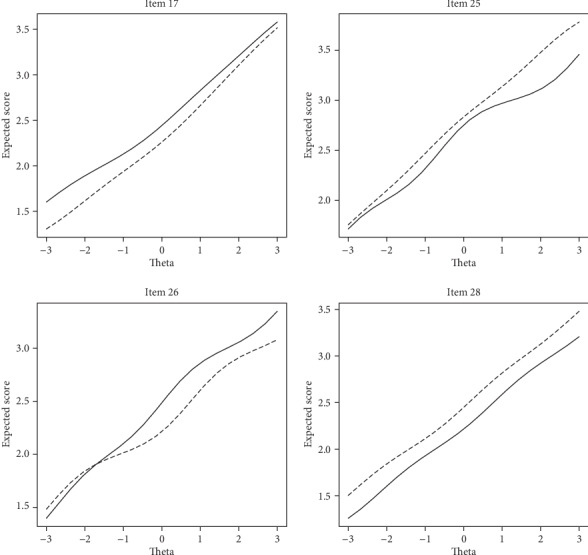
ICF of items with uniform DIF by gender, male (solid line) and female (dashed line).

**Figure 3:**
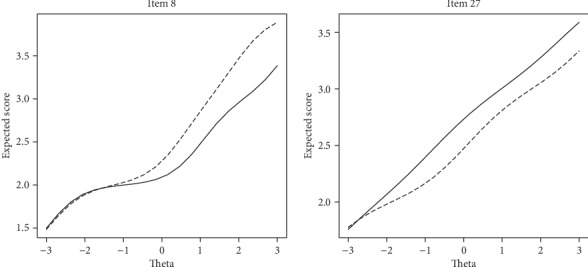
ICF of the items with uniform DIF by marital status, single (solid line) and married (dashed line).

Results indicate that four items of OHI show uniform DIF across gender and two items with uniform DIF across the marital status.  Table 4 represents summary results for assessing DIF across gender. Note that, for example, *p*_12_ refers to the observed significance level for comparing models 1 and 2. In the same way, *∆R*_12_^2^ refers to the observed *R*^2^ difference between models 1 and 2. A review of the first three columns of Table 4 shows that items 17, 25, 26, and 28 have ps smaller than nominal alpha level of 0.05 (i.e., numbers in boldface). A significant difference between models 1 and 3 in addition to a nonsignificant difference between models 2 and 3 asserts a uniform DIF for items 17, 25, 26, and 28.  Figure 2 represents ICF curves for items flagged with DIF. The ICF curves for items 25 and 28 indicate that female respondents are more likely to endorse response categories corresponding to a higher level of happiness compared to male respondents. On the other hand, ICF curves for items 17 and 26 indicate that males had higher expected scores of happiness compared to females.  Table 5 shows the results of assessing DIF across the marital status. Note that, for example, *p*_12_ refers to the observed significance level for comparing models 1 and 2. In the same way, *∆R*_12_^2^ refers to the observed *R*^2^  difference between models 1 and 2. Based on the figures in Table 5, items 8 and 27 showed uniform DIF across the marital status. As shown in Figure 3, item 8 was in favor of the married participants, whereas item 27 was in favor of single individuals. On the other words, single individuals have higher expected scores of happiness compared to married participants in item 8 and vice versa in item 27. The measures of effect size show whether a statistically significant outcome (*p* < 0.05) is also practically significant or not. According to the framework to DIF effect size proposed by Jodoin and Gierl (2001), all DIF items for both DIF factors in Table 1 show negligible DIF (all effect size ≤0.035). The values of *ν*^2^ were 0.03 and -0.004 for gender and marital status, respectively. These values indicated a small effect size according to Penfield and Algina (2006). Therefore, there is not an overall bias at the test level.

## 4. Discussion

Previous studies found the OHI to be a reliable and psychologically valid tool for assessing levels of happiness among adolescents. To date, there is no study that had looked at the validity of OHI in terms of measurement invariance and potential bias with respect to previously identified groups such as gender and marital status. Because of polytomous response style of OHI, this study utilized OLR in order to assess DIF of OHI items and DFT across gender and marital status. The psychometric properties of the OHI were also examined as a prerequisite for DIF analysis. The current results showed the appropriateness of using the GRM for analyzing OHI. The measurement invariance of OHI revealed six out of 29 items of the OHI were flagged as exhibiting uniform DIF (four items across gender and two across marital status). Examination of effect sizes suggested that observed uniform DIF is practically negligible. Very low values of *ν*2 also suggested negligible differential test functioning across gender and marital status. These important findings signify the validity and fairness of OHI for assessing happiness regardless of their gender or marital status. It turned out that, although in previous studies OHI was found not to be strictly unidimensional [8, 13, 37], this had very little impact on the DIF analysis. Like other researchers, this study had some limitations, which should be taken into consideration before drawing conclusions from its results. The major limitation of the present study was that we just assessed DIF across two variables so further research is needed to fully evaluate the generalizability of the results by looking at other grouping variables such as culture, age groups, job, and education. Another potential limitation was that students from different academic programs/colleges in the present study have been treated the same. Different simulation studies have shown that ignoring the hierarchical structure of data (e.g., students nested in programs/colleges) might affect the estimated parameters of the model. It has been mentioned that choosing proper modeling in analyzing hierarchical data is crucial as it allows for a potentially greater understanding of the issue under study, as well as avoiding statistical misspecification [11, 20, 38]. Therefore, the hierarchical OLR (HOLR) model should also be used in future studies for nested data. In conclusion, this study was a significant step towards providing theoretical and practical information regarding the assessment of happiness by presenting adequate evidence regarding the psychometric properties of OHI. Future studies may look at different methods for assessing DIF and different groups for strengthening conclusions with respect to OHI.

## Figures and Tables

**Table 1:** Goodness of fit statistics of different IRT models.

Model	M2	Degrees of freedom	*p* value	RMSEA	RMSEA-5%	RMSEA-95%	TLI	CFI
GRM	1144.054	319	<0.001	0.072	0.067	0.076	0.927	0.933
GPCM	1166.433	319	<0.001	0.073	0.068	0.077	0.925	0.931
RSM	1572.113	403	<0.001	0.076	0.072	0.08	0.918	0.905

Notes: RMSEA: root mean square of error approximation; TLI: Tucker-Lewis index; CFI: comparative fit index.

**Table 2:** Test statistics of comparison of different IRT models.

	RSM	GPCM	GRM
RSM	-14402.06		
GPCM	1158.254∗∗	-13822.93	
GRM	1319.515∗∗	161.261^∗∗^	-13742.30

Notes: ∗∗*p* value ≤0.001; Main diagonal: log-likelihood values; Off-diagonal: chi-square test of model comparison.

**Table 3:** GRM item parameter estimate and fit statistics for 29 of OHI.

Items content	a	b1	b2	b3	S-X^2^
Q1	1.724	-1.260	0.885	2.73	70.511
Q2	1.43	-1.887	0.341	1.491	73.791
Q3	1.862	-2.738	-0.243	2.25	47.594
Q4	1.498	-1.687	0.228	2.446	49.494
Q5	2.436	-0.896	0.993	2.239	48.539
Q6	1.992	-0.7	0.917	2.189	50.641
Q7	1.846	-2.805	-0.78	2.198	41.504
Q8	2.24	-1.331	0.446	1.325	66.185
Q9	0.995	-3.176	0.126	3.108	60.153
Q10	1.11	-1.355	0.701	3.108	83.775
Q11	1.05	-1.027	1.186	2.576	98.627
Q12	0.953	-1.513	0.257	3.793	64.521
Q13	1.687	-1.978	0.424	2.393	56.818
Q14	1.73	-2.525	-0.504	1.941	38.113
Q15	1.353	-1.798	0.316	1.878	86.021
Q16	1.525	-2.26	0.164	1.702	52.78
Q17	1.087	-2.155	0.578	2.382	71.709
Q18	1.272	-1.737	-0.022	3.102	75.179
Q19	2.143	-2.404	0.146	2.097	30.131
Q20	1.87	-0.943	0.608	2.686	59.746
Q21	1.452	-1.02	0.956	2.961	88.409^∗^
Q22	1.285	-3.056	-0.744	1.682	65.058
Q23	1.348	-2.971	-0.557	2.365	62.571
Q24	1.928	-2.212	0.16	1.471	62.383
Q25	1.356	-2.567	-0.625	1.584	69.014
Q26	1.714	-1.694	0.345	2.262	53.011
Q27	1.153	-3.176	-0.517	2.332	52.344
Q28	1.236	-2.165	0.229	2.465	85.217
Q29	1.862	-2.628	0.17	1.865	46.971

Notes: ∗*p* value ≤0.05, a: item discrimination; b: item difficulty threshold; and S-X^2^: item fit values.

**Table 4:** Results of assessing DIF across gender.

Item	*p*_13_	*p*_12_	*p*_23_	*∆R*_13_^2^	*∆R*_12_^2^	*∆R*_23_^2^	Type of DIF	Magnitude
Q1	0.943	0.189	0.625	0.002	0.000	0.001	NO	—
Q2	0.496	0.615	0.389	0.000	0.000	0.000	NO	—
Q3	0.907	0.814	0.84	0.000	0.000	0.0000	NO	—
Q4	0.499	0.633	0.577	0.000	0.000	0.000	NO	—
Q5	0.355	0.426	0.463	0.001	0.000	0.000	NO	—
Q6	0.373	0.49	0.266	0.001	0.001	0.000	NO	—
Q7	0.791	0.251	0.876	0.001	0.000	0.001	NO	—
Q8	0.098	0.647	0.112	0.002	0.002	0.000	NO	—
Q9	0.627	0.632	0.507	0.000	0.000	0.000	NO	—
Q10	0.337	0.551	0.412	0.000	0.000	0.000	NO	—
Q11	0.457	0.207	0.688	0.001	0.000	0.001	NO	—
Q12	0.671	0.492	0.509	0.000	0.000	0.000	NO	—
Q13	0.07	0.578	0.084	0.003	0.003	0.000	NO	—
Q14	0.972	0.597	0.885	0.000	0.000	0.000	NO	—
Q15	0.544	0.554	0.65	0.000	0.000	0.000	NO	—
Q16	0.747	0.283	0.503	0.001	0.000	0.001	NO	—
Q17	0.023	0.559	0.011	0.006	0.005	0.000	Uniform	Negligible
Q18	0.285	0.178	0.5	0.002	0.000	0.002	NO	—
Q19	0.128	0.455	0.067	0.004	0.003	0.000	NO	—
Q20	0.458	0.977	0.446	0.000	0.000	0.000	NO	—
Q21	0.183	0.424	0.103	0.003	0.002	0.0005	NO	—
Q22	0.844	0.278	0.573	0.001	0.000	0.001	NO	—
Q23	0.414	0.121	0.738	0.003	0.000	0.002	NO	—
Q24	0.623	0.153	0.932	0.002	0.000	0.002	NO	—
Q25	0.052	0.191	0.014	0.007	0.005	0.001	Uniform	Negligible
Q26	0.003	0.135	0.009	0.008	0.006	0.002	Uniform	Negligible
Q27	0.153	0.295	0.248	0.002	0.000	0.001	NO	—
Q28	0.003	0.795	0.002	0.009	0.009	0.000	Uniform	Negligible
Q29	0.451	0.199	0.705	0.002	0.000	0.001	NO	—

**Table 5:** Results of assessing DIF across marital status.

Item	*p*_13_	*p*_12_	*p*_23_	*∆R*_13_^2^	*∆R*_12_^2^	*∆R*_23_^2^	Type of DIF	Magnitude
Q1	0.7	0.075	0.881	0.003	0.000	0.003	NO	—
Q2	0.748	0.64	0.633	0.000	0.000	0.000	NO	—
Q3	0.896	0.216	0.783	0.002	0.000	0.002	NO	—
Q4	0.1	0.409	0.142	0.002	0.002	0.000	NO	—
Q5	0.553	0.479	0.409	0.001	0.000	0.000	NO	—
Q6	0.871	0.302	0.909	0.000	0.000	0.000	NO	—
Q7	0.869	0.131	0.698	0.003	0.000	0.003	NO	—
Q8	0.021	0.396	0.031	0.004	0.004	0.000	Uniform	Negligible
Q9	0.198	0.406	0.275	0.002	0.001	0.000	NO	—
Q10	0.404	0.712	0.447	0.000	0.000	0.000	NO	—
Q11	0.681	0.393	0.854	0.000	0.000	0.000	NO	—
Q12	0.067	0.206	0.126	0.003	0.002	0.001	NO	—
Q13	0.647	0.703	0.549	0.000	0.000	0.000	NO	—
Q14	0.515	0.509	0.643	0.000	0.000	0.004	NO	—
Q15	0.320	0.959	0.291	0.000	0.000	0.000	NO	—
Q16	0.779	0.754	0.842	0.000	0.000	0.000	NO	—
Q17	0.608	0.093	0.295	0.003	0.000	0.002	NO	—
Q18	0.187	0.492	0.243	0.001	0.001	0.000	NO	—
Q19	0.183	0.521	0.232	0.002	0.001	0.000	NO	—
Q20	0.347	0.57	0.254	0.001	0.001	0.002	NO	—
Q21	0.689	0.449	0.527	0.000	0.000	0.000	NO	—
Q22	0.424	0.108	0.746	0.002	0.000	0.002	NO	—
Q23	0.371	0.708	0.286	0.001	0.001	0.000	NO	—
Q24	0.244	0.719	0.267	0.001	0.001	0.000	NO	—
Q25	0.387	0.233	0.595	0.001	0.000	0.001	NO	—
Q26	0.536	0.94	0.503	0.000	0.000	0.000	NO	—
Q27	0.016	0.817	0.009	0.006	0.006	0.000	Uniform	Negligible
Q28	0.876	0.720	0.961	0.000	0.000	0.000	NO	—
Q29	0.826	0.585	0.686	0.000	0.000	0.000	NO	—

## Data Availability

The data used to support the findings of this study are available from the corresponding author upon request.
